# Supplementary figures and images for: Highly accurate protein structure prediction-based virtual docking pipeline accelerating the identification of anti-schistosomal compounds
Source: PLoS Pathog. 2025 Oct 13;21(10):e1013274. doi: 10.1371/journal.ppat.1013274 (PMC12533970; doi:10.1371/journal.ppat.1013274)

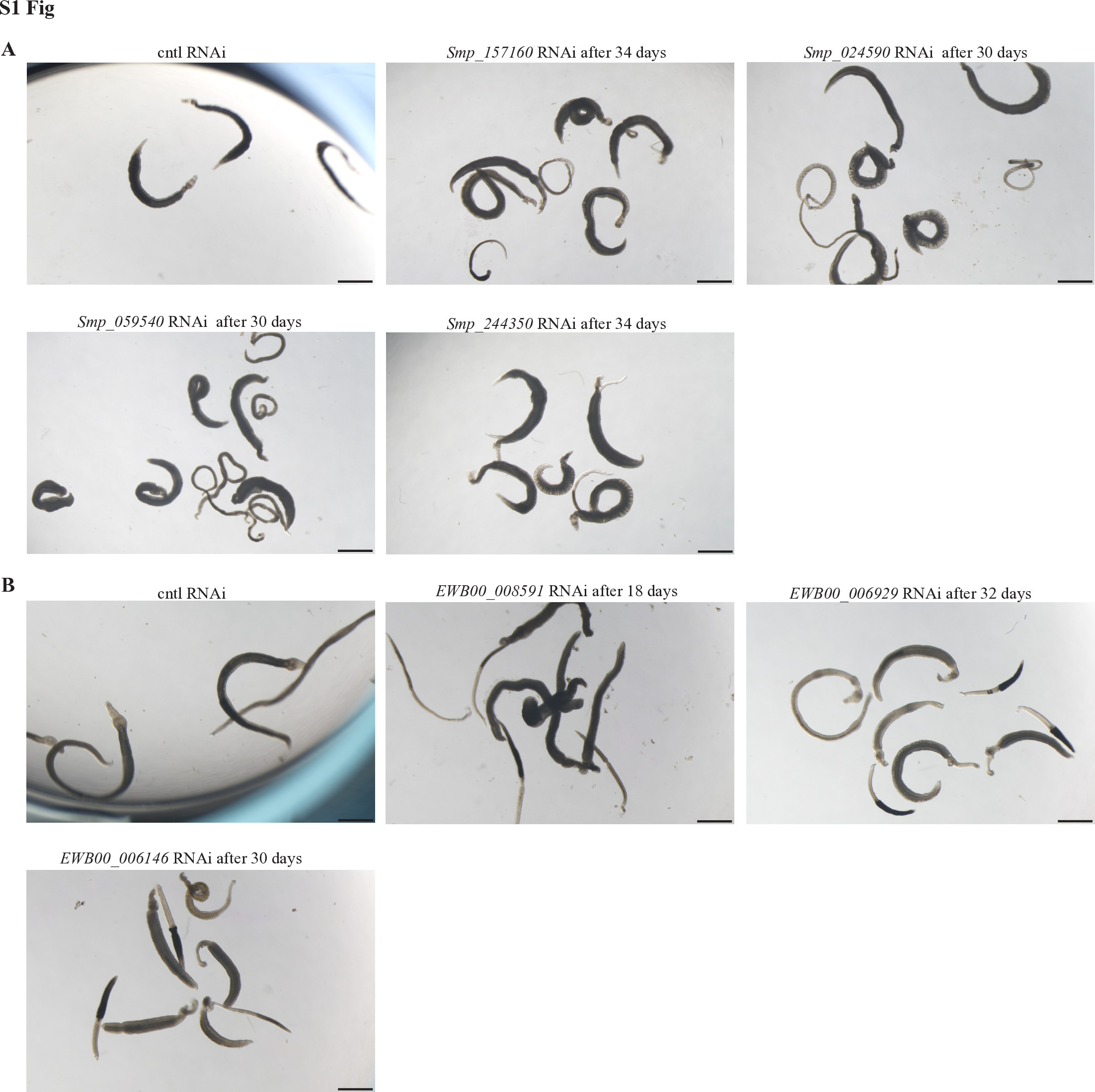

Supplement: S1 Fig — (A) Bright-field images showing phenotypes of adult S. mansoni after RNAi treatment. (B) Bright-field images showing phenotypes of adult S. japonicum after RNAi treatment. Representative images from 3 biological replicates, Scale bars, 1000 μm. (TIF) [file ppat.1013274.s001.tif]

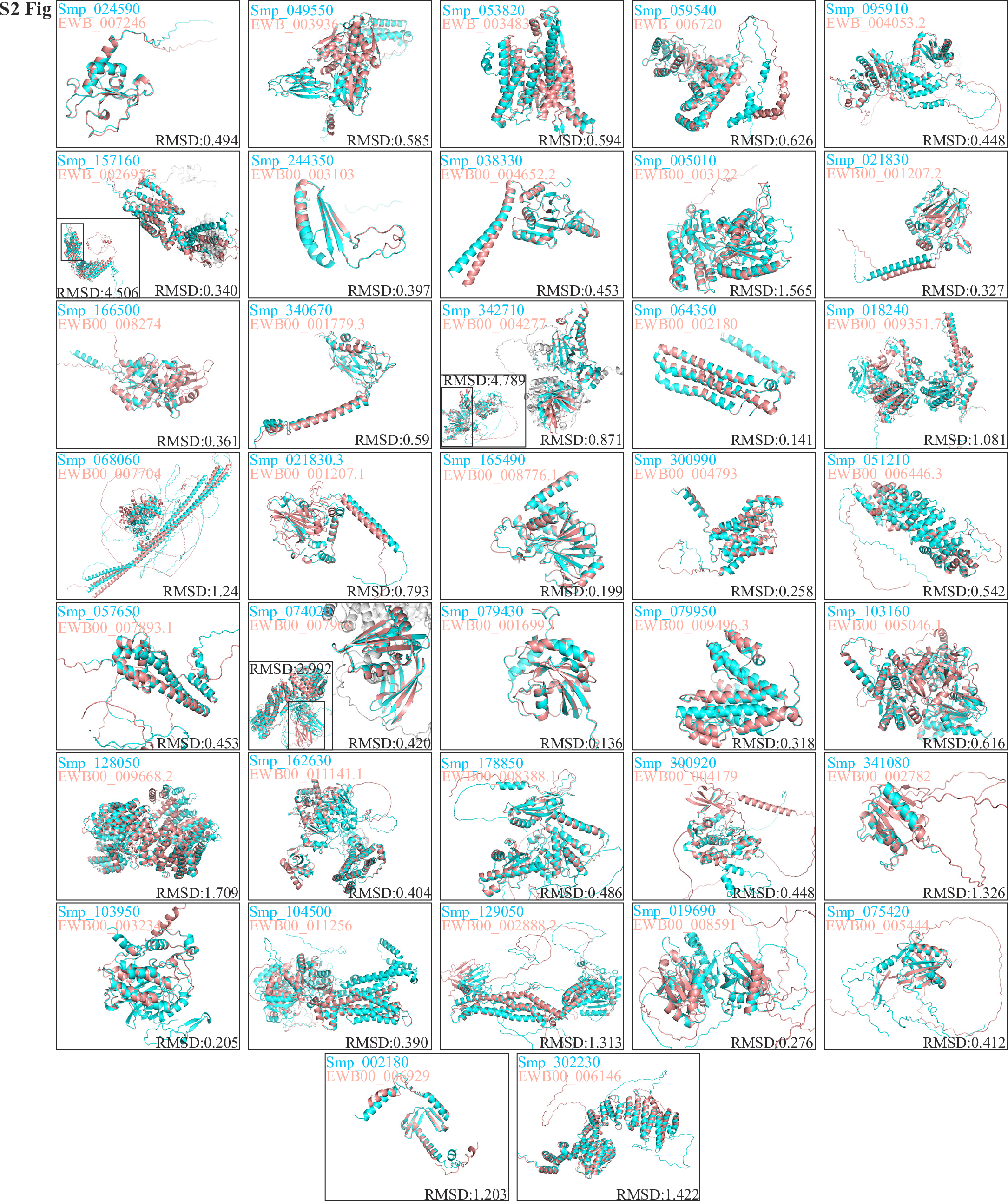

Supplement: S2 Fig — Each alignment demonstrates the structural similarity between homologous proteins from the two schistosome species. Protein structures were superimposed using PyMOL, and RMSD values were calculated to quantify structural similarity. The protein structure of S. mansoni is shown in cyan, while that of S. japonicum is depicted in salmon. The small inset in the lower left corner represents the alignment of the full-length protein structures, whereas the enlarged section highlights the alignment of conserved regions or specific protein domains. (TIF) [file ppat.1013274.s002.tif]

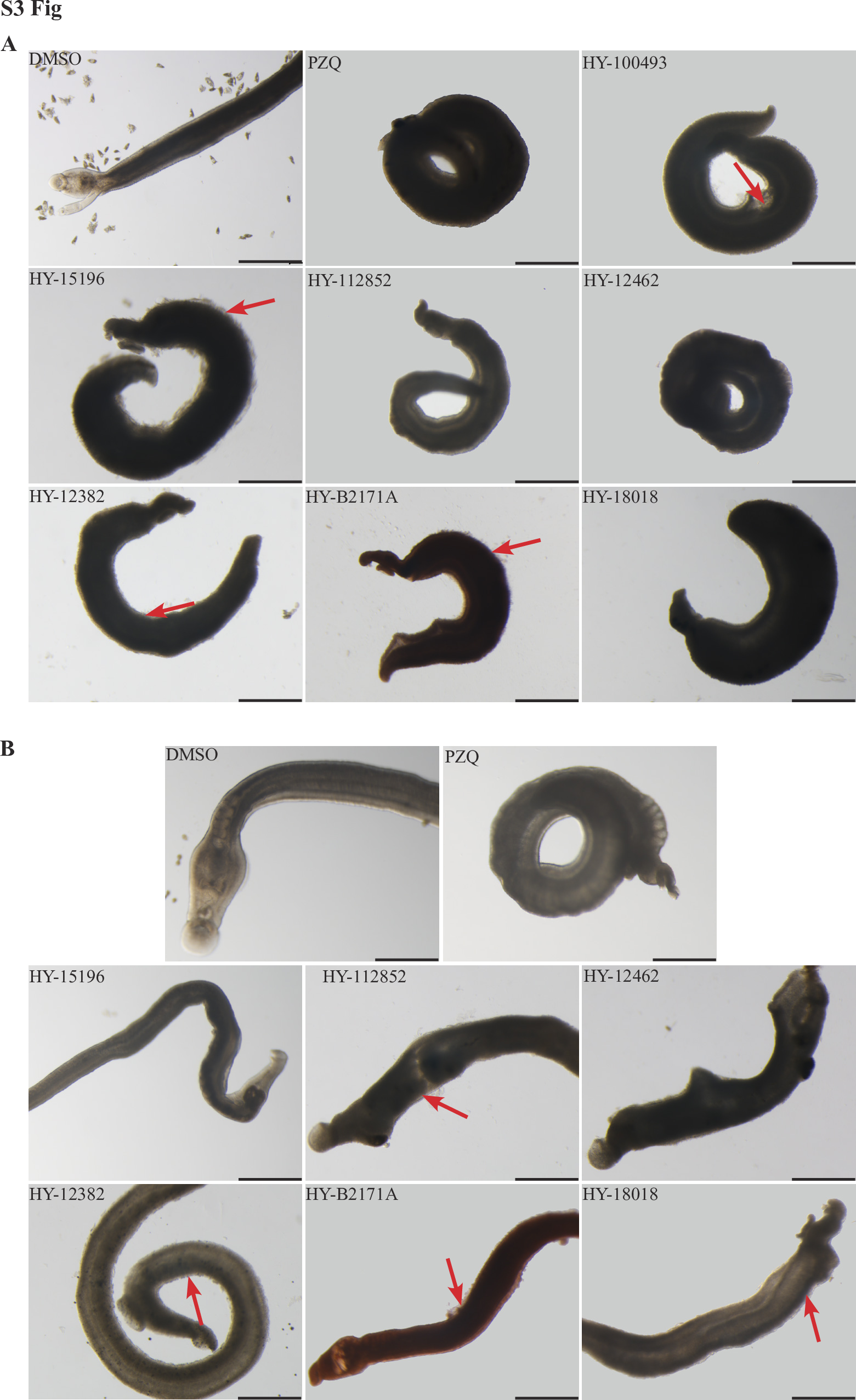

Supplement: S3 Fig — (A) Phenotypes of S. mansoni treated with different compounds at concentration of 10 μM for 7 days. (B) Phenotypes of S. japonicum treated with different compounds at concentration of 10 μM for 7 days. DMSO as negative control; PZQ as positive control. Scale bars, 500 μm. Red arrows denote tegumental erosion. (TIF) [file ppat.1013274.s003.tif]

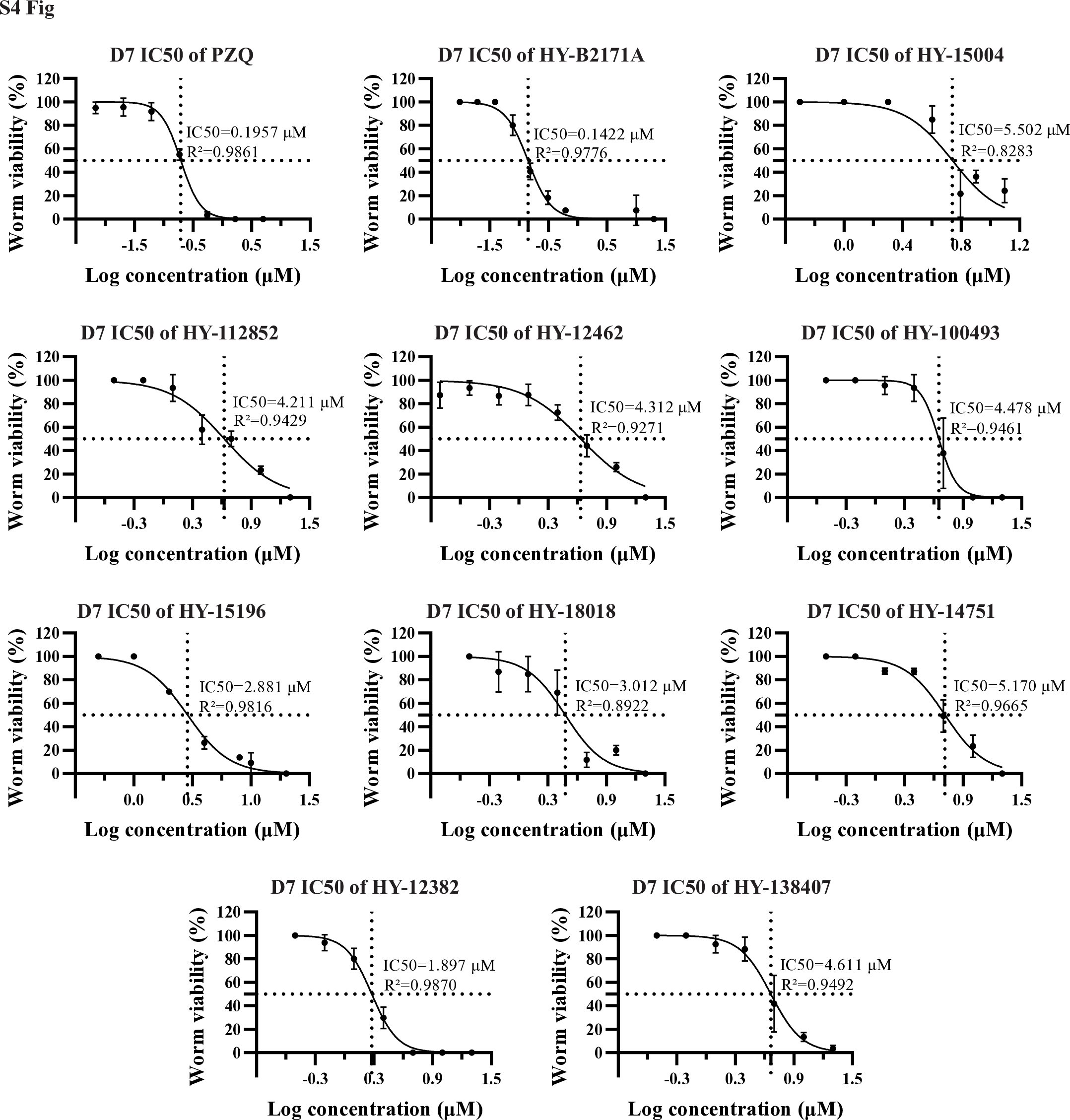

Supplement: S4 Fig — IC50 values were determined for ten high-efficacy compounds targeting adult S. mansoni. The fitted IC50 curves are shown in the figure, with praziquantel (PZQ) used as a control. Dose-response curves were generated using GraphPad Prism based on IC50 values calculated from experimental data. The IC50 value was evaluated on Day 7 (D7) post-treatment. (TIF) [file ppat.1013274.s004.tif]

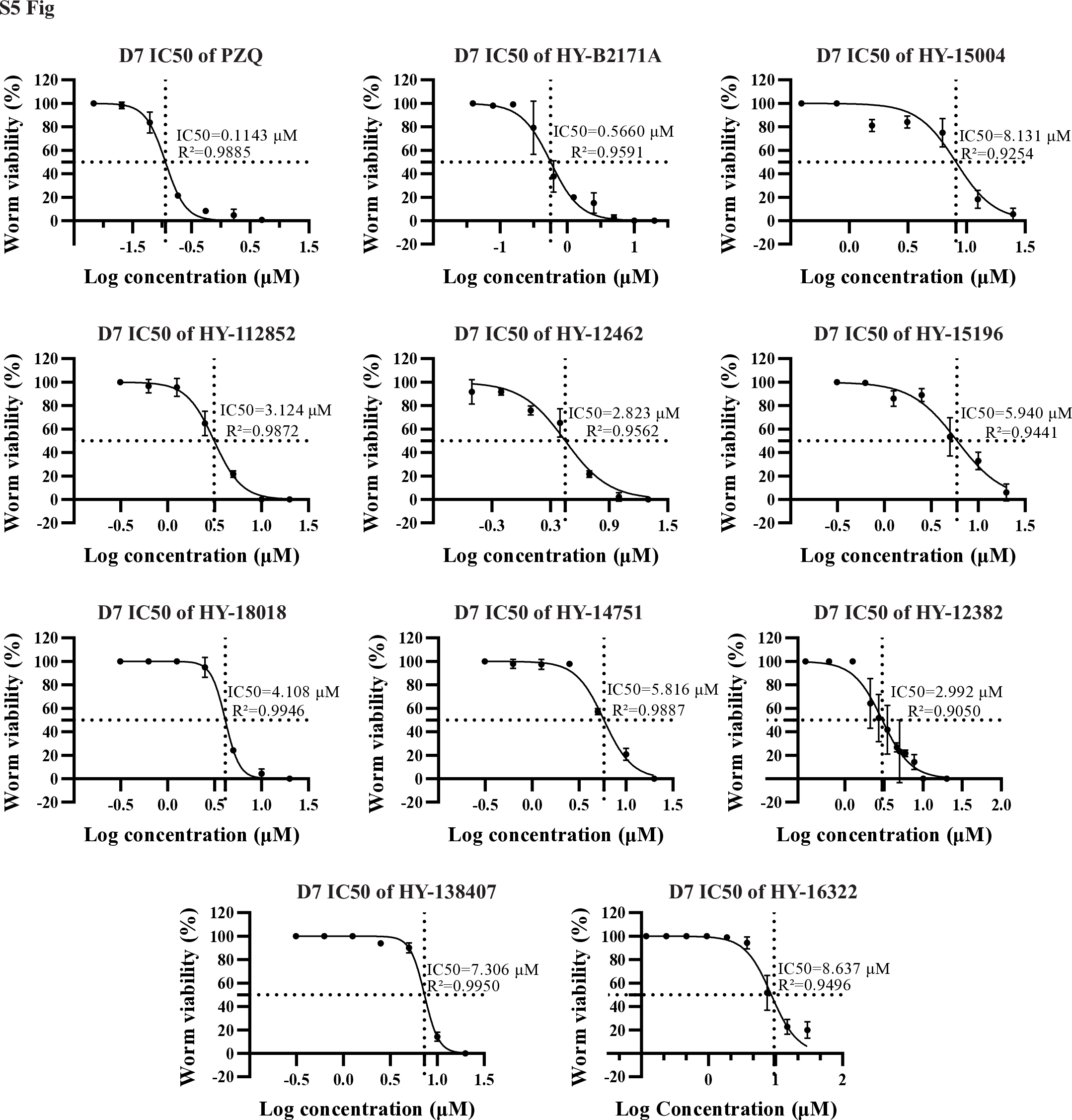

Supplement: S5 Fig — IC50 values were determined for ten high-efficiency compounds targeting adult S. japonicum., The fitted IC50 curves are shown in the figure, with praziquantel (PZQ) used as a control. Dose-response curves were generated using GraphPad Prism based on IC50 values calculated from experimental data. The IC50 value was evaluated on Day 7 (D7) post-treatment. (TIF) [file ppat.1013274.s005.tif]

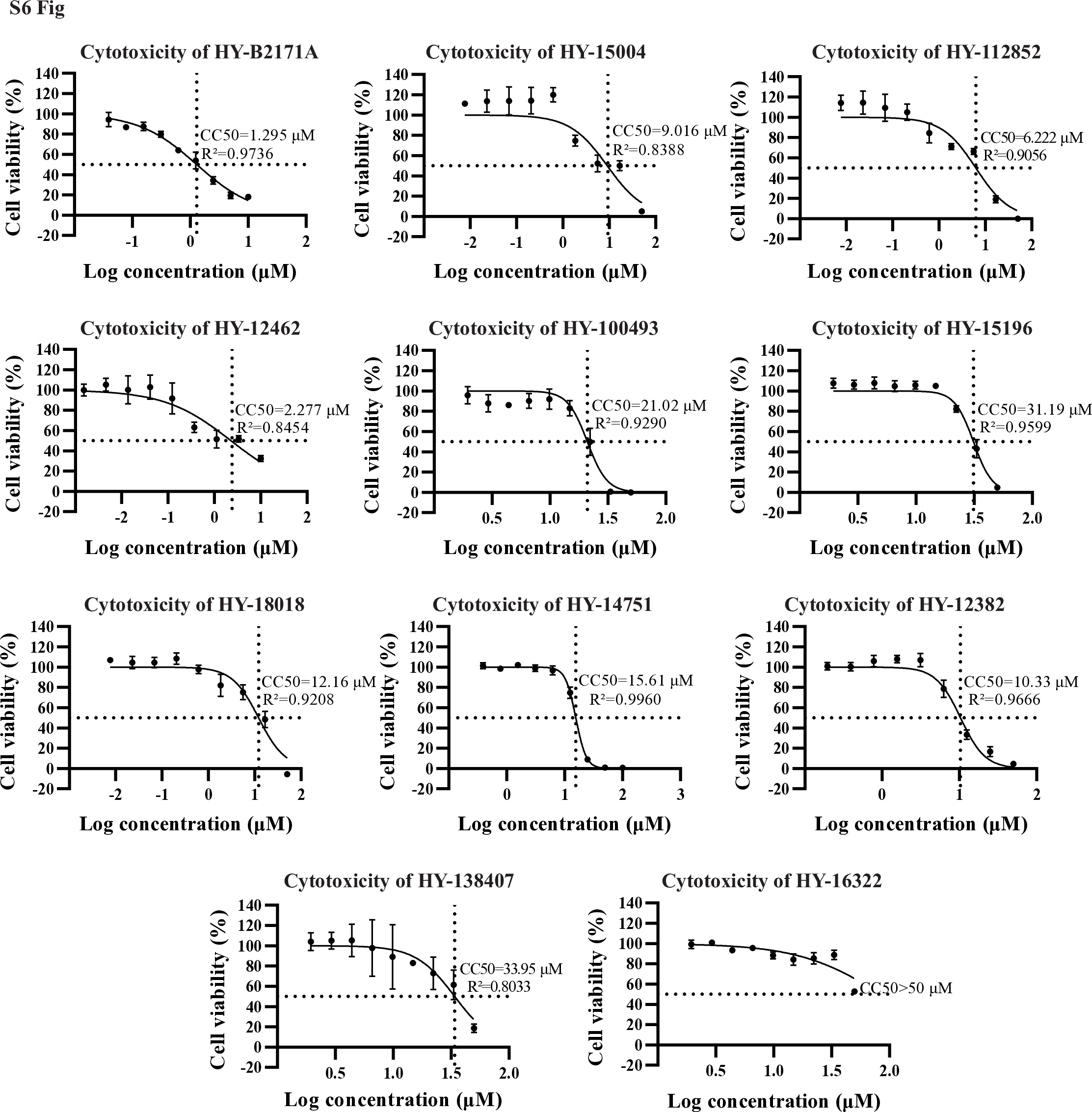

Supplement: S6 Fig — Cytotoxicity of 11 potent anti-schistosomal compounds was assessed in HepG2 cells using the CCK-8 assay. Dose–response curves were fitted using a four-parameter logistic (4PL) regression model in GraphPad Prism software. (TIF) [file ppat.1013274.s006.tif]

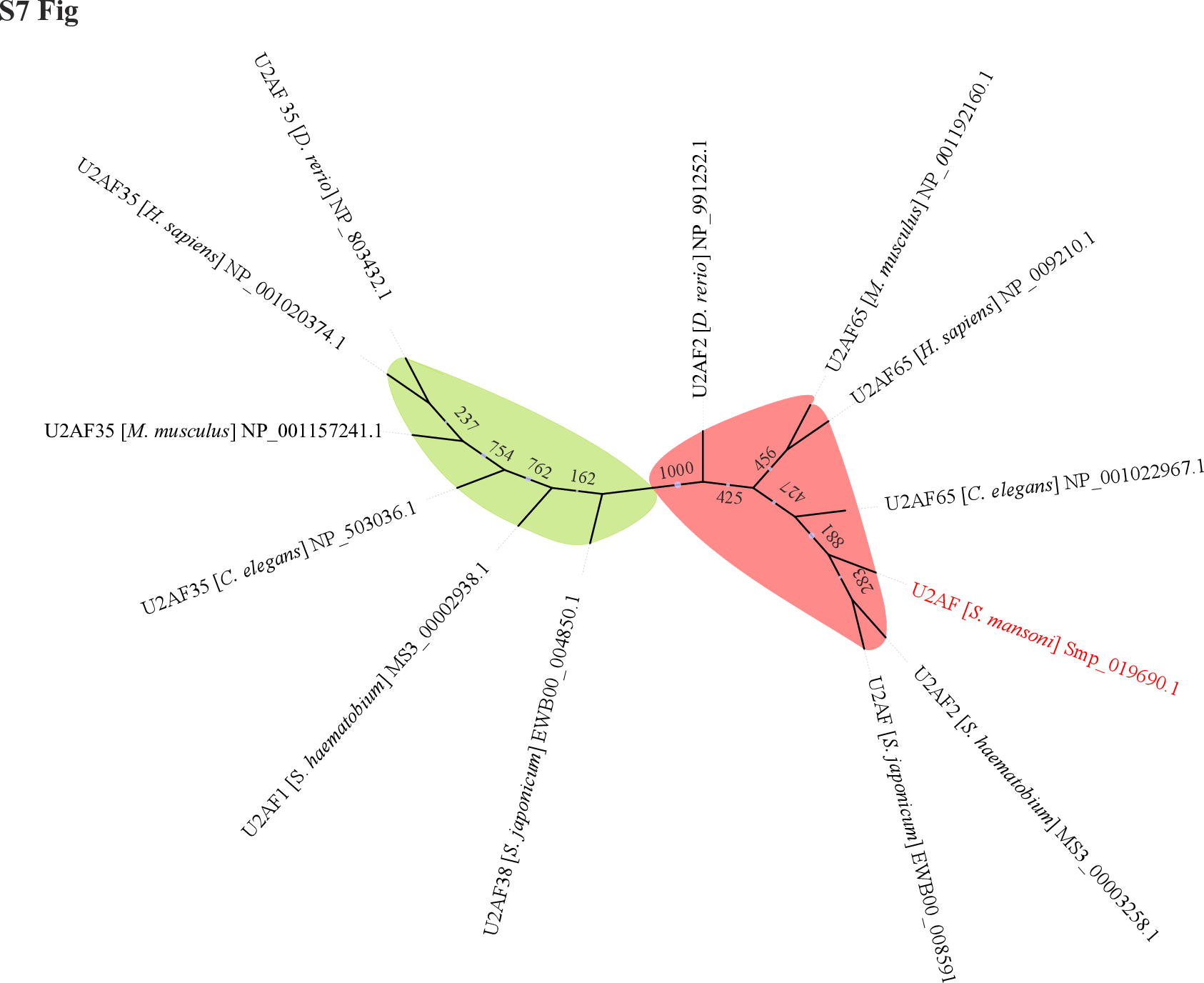

Supplement: S7 Fig — The green leaf represents U2AF35, the red leaf represents U2AF65. The tree was constructed using the Maximum Likelihood method in MEGA 11, based on amino acid sequences from 7 species. Bootstrap values from 1000 replicates are indicated at the nodes. Smp_019690 belongs to the U2AF65 family. (TIF) [file ppat.1013274.s007.tif]

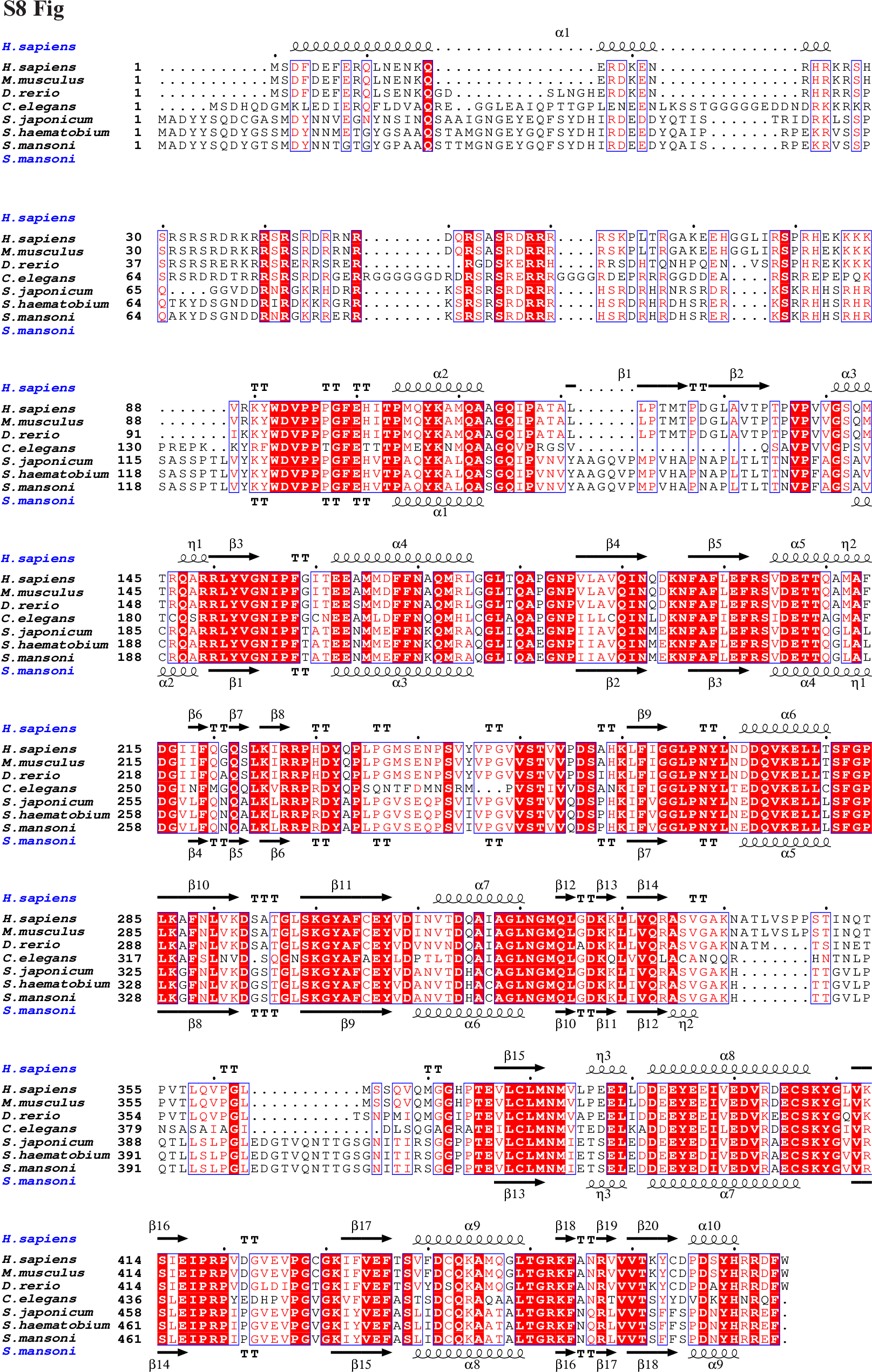

Supplement: S8 Fig — Multiple sequence alignment of U2AF65 proteins across various species, including S. mansoni (Smp_019690.1), S. haematobium (MS3_00003258.1), S. japonicum (EWB00_008591), Caenorhabditis elegans (NP_001022967.1), Danio rerio (NP_991252.1), Mus musculus (NP_001192160.1), and Homo sapiens (NP_009210.1). The percentage of conserved residues was calculated for each column based on physicochemical properties. Columns with a similarity score above 0.7 are considered highly conserved, with residues highlighted in red and framed in blue. Strictly identical residues appear in white on a red background. The blue-font-labeled line above the alignment represents the predicted secondary structure of human U2AF65, while the blue-font-labeled line below the alignment corresponds to that of S. mansoni U2AF65. (TIF) [file ppat.1013274.s008.tif]

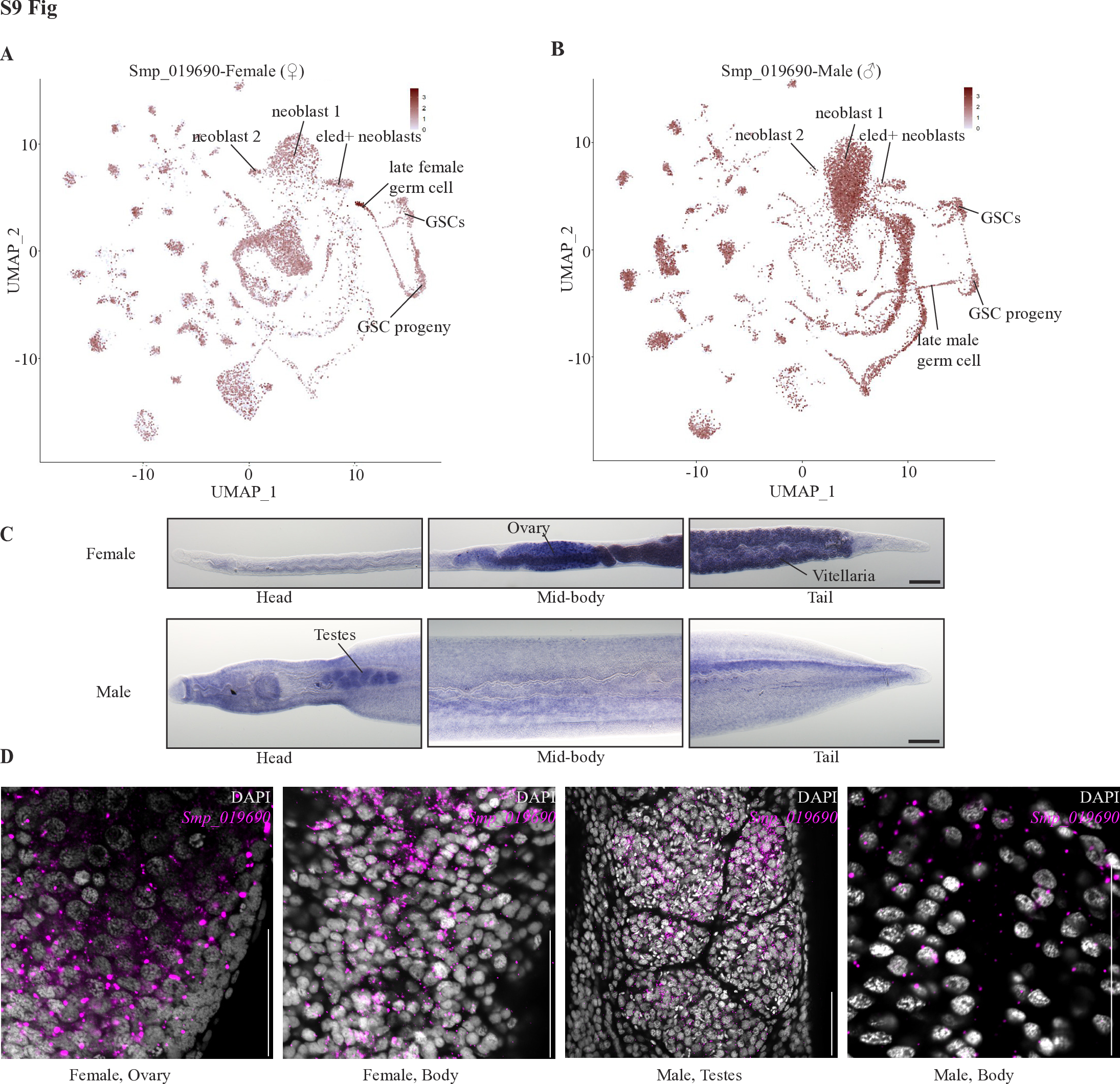

Supplement: S9 Fig — (A, B) UMAP plots showing the expression pattern of Smp_019690 in adult female (A) and adult male (B) S. mansoni, obtained from the S. mansoni single-cell atlas (https://www.collinslab.org/schistocyte/). Color intensity represents the expression levels, with darker shades indicating higher expression. (C) WISH showing expression of Smp_019690 in adult female and male worms. Representative images from n > 5 worms. Scale bars, 100 μm. (D) FISH showing expression of Smp_019690 in somatic tissues and reproductive organs of adult female and male worms. Representative images from n > 5 worms. Nuclei are labeled by DAPI (grays), Smp_019690 mRNA shown in magenta. Scale bars, 50 μm. (TIF) [file ppat.1013274.s009.tif]

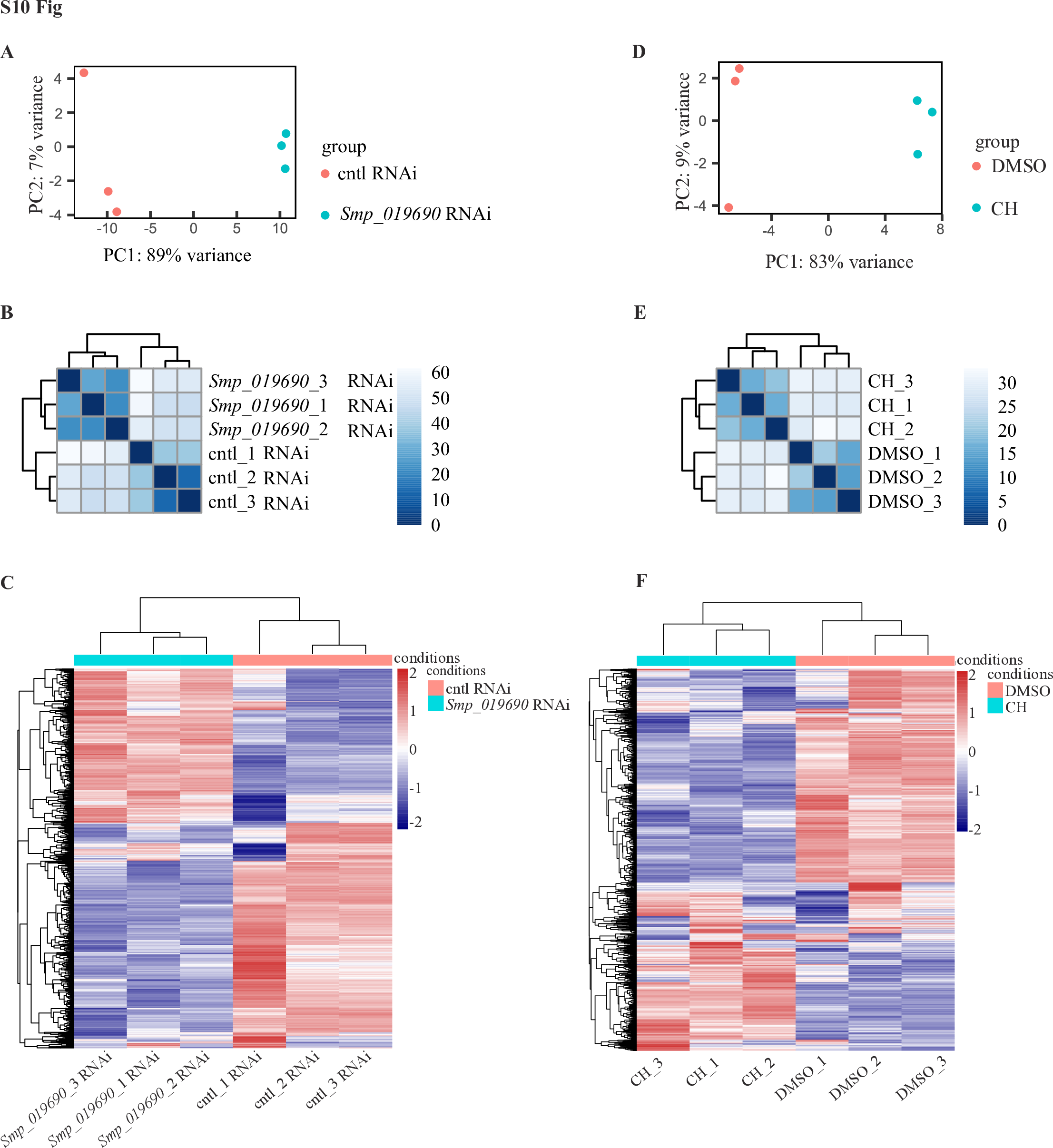

Supplement: S10 Fig — (A) Principal Component Analysis (PCA) on the RNA-seq data under Smp_019690 RNAi. (B) Heatmap of the Euclidean distance matrix with hierarchical clustering of transcriptomes from Smp_019690 RNAi and control RNAi samples. (C) Heatmap showing the cluster of top 2000 highly variable genes in the parasites from Smp_019690 RNAi and control group. (D) PCA on the RNA-seq data under CH treatment. (E) Heatmap of the Euclidean distance matrix with hierarchical clustering of transcriptomes from CH and DMSO treatment samples. (F) Heatmap showing the cluster of top 2000 highly variable genes in the parasites from CH and DMSO treatment group. (TIF) [file ppat.1013274.s010.tif]

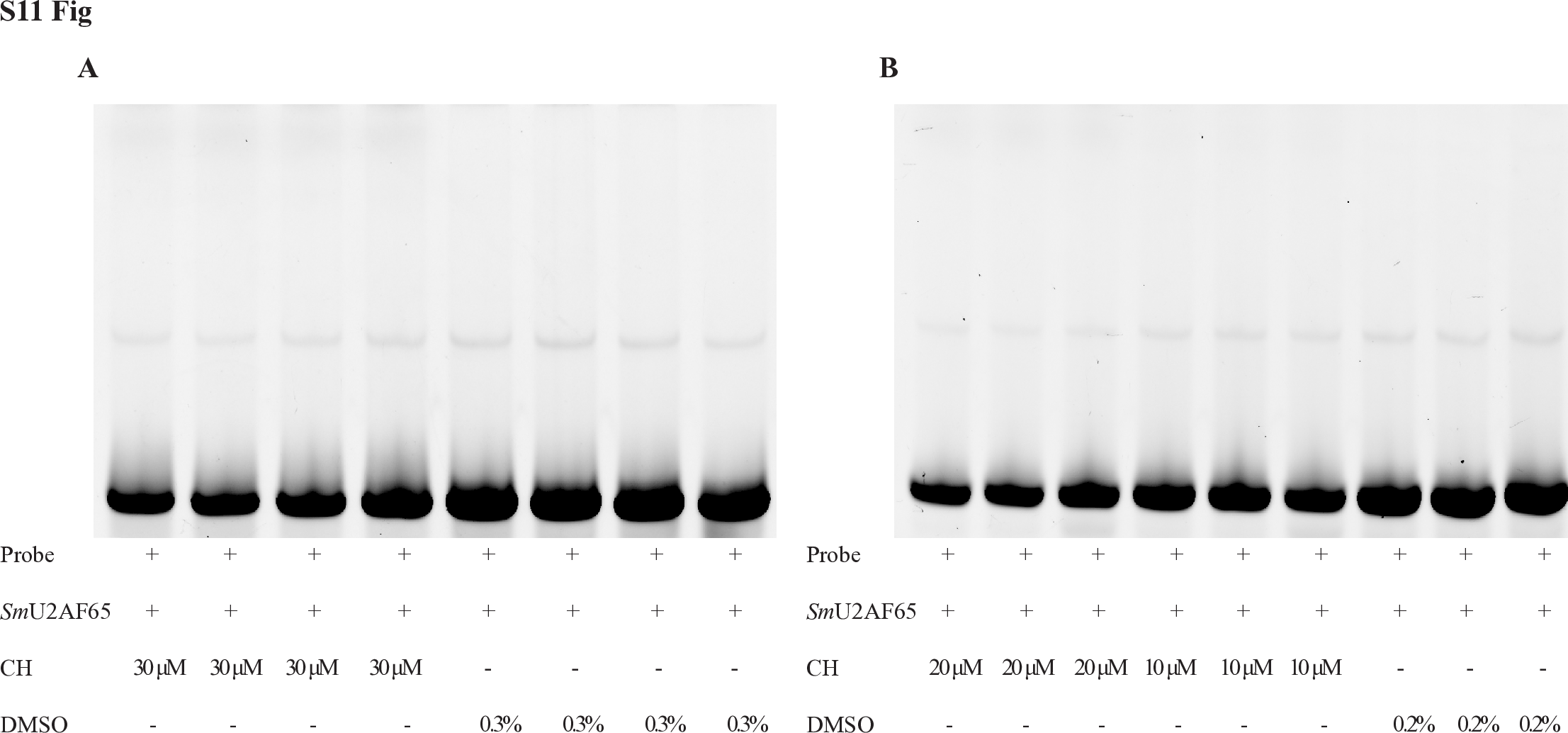

Supplement: S11 Fig — (A, B) Effects of varying concentrations of CH on the binding interaction between SmU2AF65 and RNA. Grayscale intensity analysis of the shift bands was performed using ImageJ software for quantitative evaluation. (TIF) [file ppat.1013274.s011.tif]

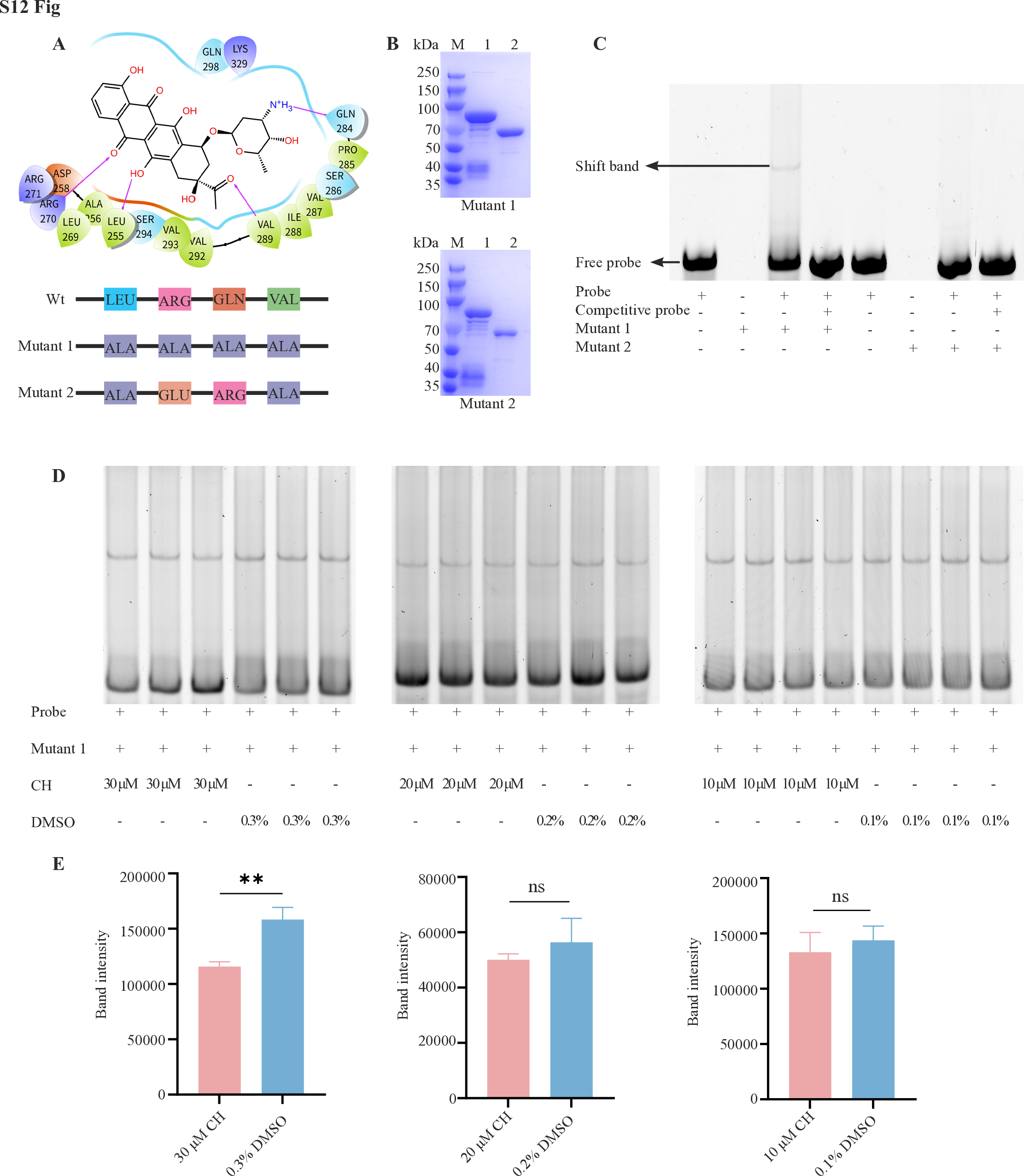

Supplement: S12 Fig — (A, upper panel) Predicted 2D interaction diagram of SmU2AF65 with CH, showing four key residues (LEU255, ARG270, GLN284, and VAL289) at the binding interface. (A, lower panel) Schematic representations of wild-type (WT) SmU2AF65 and two mutants: Mutant 1 (L255A/R270A/Q284A/V289A) and Mutant 2 (L255A/R270E/Q284R/V289A). (B) SDS-PAGE analysis of purified mutant SmU2AF65 proteins. Lane M: protein molecular weight marker; Lane 1: purified mutant protein prior to His and SUMO tag cleavage; Lane 2: purified protein after His and SUMO tag removal. (C) EMSA results showing RNA-binding activity of Mutant 1 and Mutant 2. (D) EMSA assessing the effects of increasing CH concentrations on the interaction between Mutant 1 and RNA. (E) Quantitative analysis of Mutant 1–RNA shift band intensity at different CH concentrations, based on grayscale measurements from panel D using ImageJ. **P < 0.01, ns, P > 0.05. (TIF) [file ppat.1013274.s012.tif]

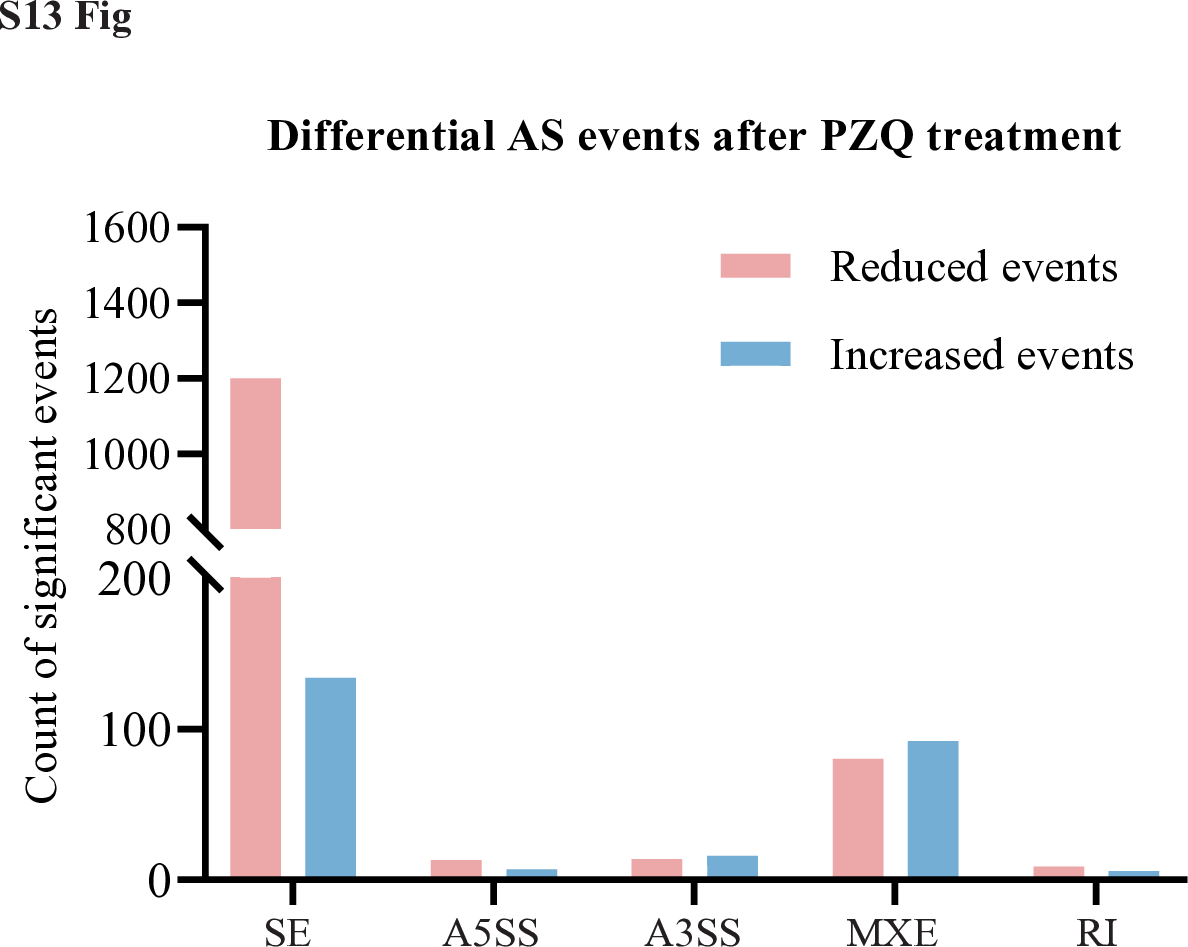

Supplement: S13 Fig — Differential alternative splicing events after 0.3 μM PZQ treatment for 12 hours. AS, alternative splicing; SE, skipped exon; A5SS, alternative 5' splice sites; A3SS, alternative 3' splice sites; MXE, mutually exclusive exons; RI, retained intron. (TIF) [file ppat.1013274.s013.tif]

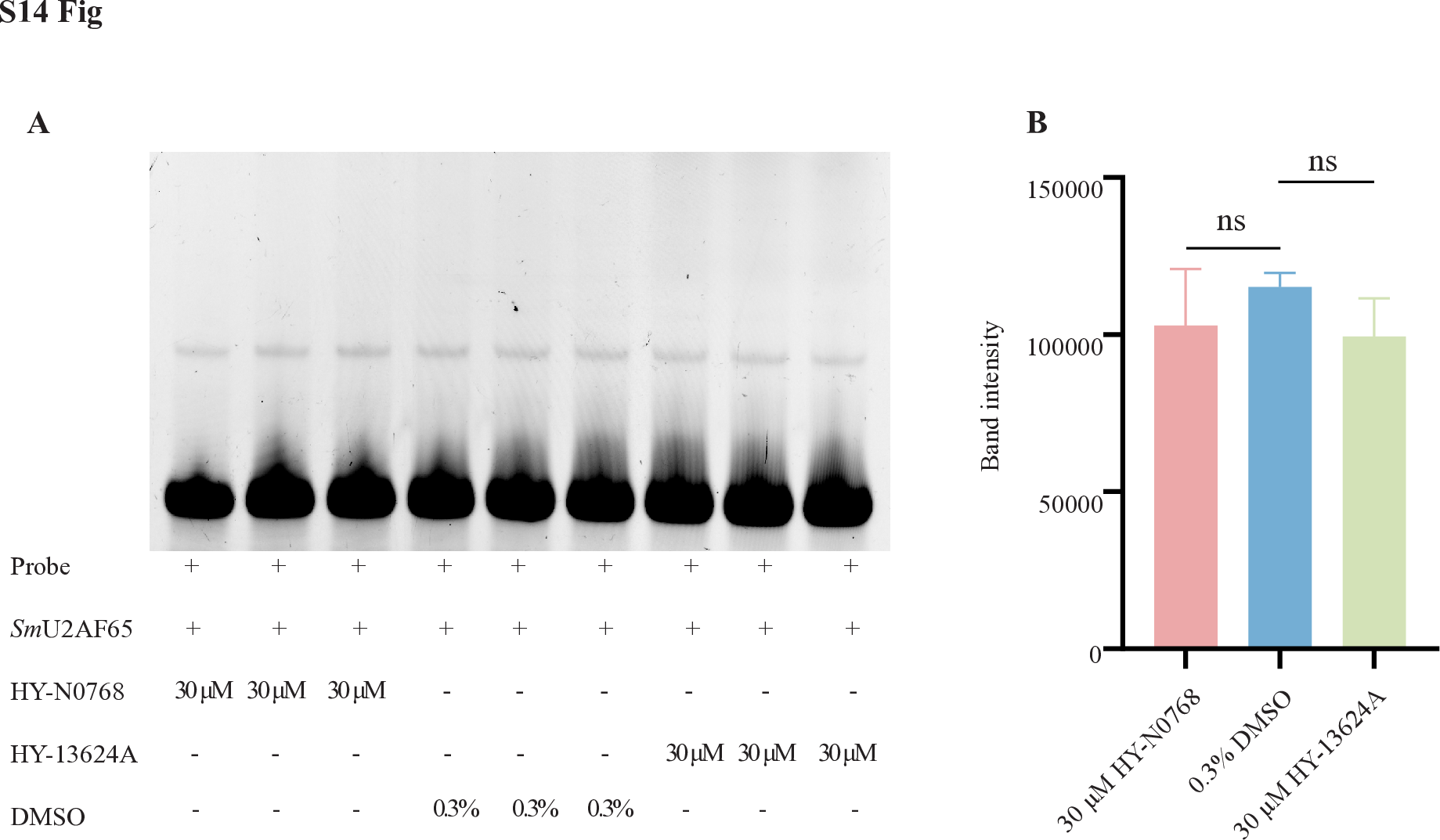

Supplement: S14 Fig — (A) Representative EMSA gel depicting the binding of recombinant SmU2AF65 protein to RNA in the presence of 30 μM HY-N0768 or HY-13624A. No appreciable disruption of the SmU2AF65–RNA complex was observed relative to the DMSO vehicle control. (B) Quantitative analysis of band intensities from 3 independent replicates. ns, P > 0.05. (TIF) [file ppat.1013274.s014.tif]

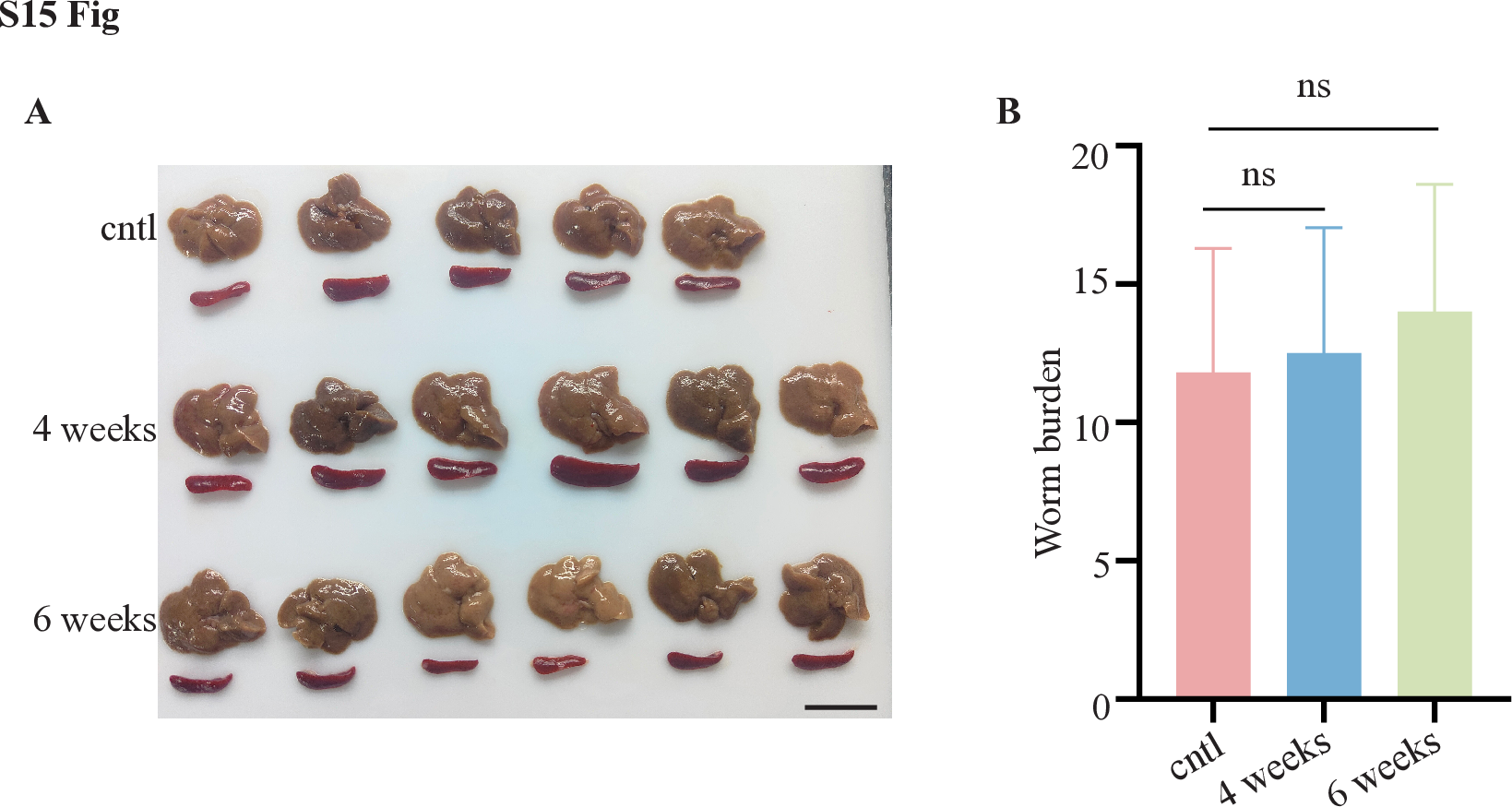

Supplement: S15 Fig — (A) Images of liver and spleen tissues from control and CH-treated mice following oral administration of CH (720 µg/kg) starting from 4 or 6 weeks post-infection. (B) Worm burden analysis in control and CH-treated groups under the same dosing regimen (mean ± SD, n ≥ 5). No significant difference was observed compared with control (ns, P > 0.05). cntl, control. Scale bars, 2 cm. (TIF) [file ppat.1013274.s015.tif]
